# Blood-Based Biomarkers for Alzheimer Disease in Primary Care: The Gap Between Biology and Clinical Utility

**DOI:** 10.1212/WNL.0000000000218403

**Published:** 2026-08-12

**Authors:** Jemma Hazan, Simone Salemme, Kathy Y. Liu, Robert Howard

**Affiliations:** 1 Division of Psychiatry, University College London, United Kingdom;; 2 North London NHS Foundation Trust, United Kingdom;; 3 Department of Biomedical, Metabolic and Neural Sciences, University of Modena and Reggio Emilia, Italy; and; 4 International School of Advanced Studies, University of Camerino, Italy.

## Abstract

Blood-based biomarkers (BBMs) for Alzheimer disease (AD) have accelerated the prospect of earlier and more accessible detection of AD pathology, potentially beyond specialist settings. However, their introduction in primary care raises important questions about clinical sequencing, interpretability, pathway consequences, and real-world utility. In this Personal View, we argue that the main barrier to responsible implementation is not analytical performance alone, but the absence of a sufficiently defined diagnostic infrastructure in which a test result has a clear and actionable role. In low-prevalence and clinically heterogeneous primary care populations, inadequate clinical anchoring and unstable pretest probability may undermine interpretability and promote category drift from diagnostic testing towards case-finding or quasiscreening. Moreover, the value of a rule-out strategy depends on whether a negative result meaningfully changes management. Until stronger evidence, clearer guidance, and appropriate service conditions are in place, primary care use of AD BBMs should remain selective, clinically anchored, and pathway-dependent.

## Introduction

Alzheimer disease (AD) blood-based biomarkers (BBMs) have shown high accuracy for detecting AD neuropathology and strong concordance with CSF and amyloid PET measures in research and specialist clinical settings.^1,2^ Among AD BBMs, plasma phosphorylated tau isoforms have emerged as leading candidates. Plasma p-tau217 has demonstrated strong performance for detecting AD-related amyloid and tau pathology and has been incorporated into an US Food and Drug Administration (FDA)–cleared plasma ratio assay intended to aid identification of amyloid pathology in symptomatic patients in specialized care settings.^3^ Plasma p-tau181 has also entered regulatory use. In October 2025, the first AD BBM—Elecsys Phospho-Tau (181P) Plasma—received FDA “clearance” as a class II device to aid the initial assessment of AD and other causes of cognitive decline in adults presenting with cognitive complaints, symptoms, or signs in primary care.^4^ These regulatory developments not only illustrate the accelerating clinical translation of AD BBMs but also highlight that different assays may have different intended uses, target populations, and care setting implications.^5^

Yet regulatory and clinical momentum is advancing faster than the evidentiary and implementation framework needed to support it, and formal guidance for primary care use remains lacking: the Alzheimer's Association (AA) 2022 Appropriate Use Recommendations identified prospective validation in primary care and assessment of whether BBMs improve diagnosis and clinical management as key priorities,^6^ whereas the AA 2025 Clinical Practice Guidelines address AD BBM use only in specialist settings.^7^

In this Perspective, we examine 4 interrelated questions: (1) how should BBMs be sequenced within the diagnostic process; (2) how does pretest probability affect interpretation and category drift; (3) what are the real-world pathway consequences of rule-out testing pathways; and (4) under what conditions might an AD BBM have clinical utility. We argue that the main barrier to responsible implementation in primary care is the absence of a sufficiently defined diagnostic infrastructure within which a test result has a clear and actionable role. The central challenge is the translational gap between access to biological information and clinical utility.

Although this Perspective focuses on primary care facing use of AD BBMs, the issues it raises are not solely under the control of primary care clinicians. Decisions about referral criteria, confirmatory biomarker access, treatment eligibility, and counselling require a coordinated diagnostic infrastructure involving primary care, secondary care dementia specialists, laboratories, policymakers, and health service planners. Accordingly, the intended audience of this article includes not only clinicians who may order these tests but also specialists and decision makers responsible for designing the pathways within which test results are interpreted and acted upon.

## Clinical Anchoring and Operationalization of an AD BBM

Assessment of a person presenting with a cognitive complaint should follow a structured, stepwise, comprehensive process.^8^ In primary care, available time, levels of expertise and confidence in dementia assessment will vary.^8,9^ AD BBMs should be introduced once a defined clinical threshold for a cognitive disorder has been reached, which requires 3 conditions: confirmation of objective cognitive abnormality and/or functionally relevant decline; reasonable exclusion of major reversible or treatable causes; and assignment of a working syndrome classification—mild cognitive impairment (MCI) or dementia—with at least moderate confidence.^9^ Only beyond this point does aetiologic attribution and biological subtyping become meaningfully interpretable^6,10^ and an AD BBM may add clinically useful information beyond history, examination, cognitive testing, and basic investigations.^11^

Use before this threshold risks a category error.^12^ A biomarker may indicate AD pathology, but without sufficient clinical anchoring, it cannot determine whether that pathology is causally relevant, incidental, or accompanied by alternative or mixed etiologies. In an older person with a nonspecific memory complaint but no clearly established cognitive syndrome, a positive AD BBM may appear diagnostically explanatory when its significance is uncertain, increasing rather than resolving uncertainty.

This diagnostic sequencing principle is consistent with the positioning of the 181P assay.^4^ In the FDA-cleared study, biomarker measurement was evaluated within a broader framework of structured cognitive assessment, syndrome classification and imaging. AD BBMs therefore do not replace clinical assessment, but are embedded within it. The key implementation question is not whether AD pathology can be detected earlier and less invasively, but whether this occurs at the correct point in the diagnostic sequence.

## Pretest Probability and Category Error in the Primary Care Setting

Once a cognitive impairment syndrome has been established, the next question is whether an AD BBM is likely to be interpretable and decision-relevant in that individual. This is fundamentally a question of pretest probability: the likelihood, before testing, that the person has AD pathology.^13^ In primary care, pretest probability reflects baseline AD pathology prevalence, selectivity of patients chosen for testing and the quality of the clinical assessment used to define that group.^14^ Biomarker results are not interpretable in isolation, but depend on who is tested, how they were selected, and how well the preceding clinical anchoring was performed.^11^

This issue is particularly important in primary care, often the first point of contact for people with cognitive concerns. The population is heterogeneous and includes individuals with subjective complaints, early or mild cognitive syndromes, non-neurodegenerative conditions and alternative or mixed causes of impairment. As a result, the pretest probability of AD pathology is lower than in specialist settings and is estimated to be ∼10%–25%.^15^ Even when primary care settings include clinicians with specialist expertise in dementia, the populations assessed in these settings are likely to remain broader, more heterogeneous, and characterized by lower or less stable pretest probability than those seen in highly specialized memory clinics, thereby limiting the operational value of a rule-out strategy. The FDA-cleared 181P study enrolled adults aged 55–80 years with “signs, symptoms, or complaints of cognitive decline,” which included a substantial proportion of subjective cognitive decline and MCI cases and a 13.1% amyloid PET positivity.^4^ Such broad inclusion criteria may support regulatory positioning, but highlight how easily the tested population can extend beyond a narrowly defined syndrome-based diagnostic group.

In this context, AD BBMs are often clinically framed as either triage tools or confirmatory tests.^16^ A triage, or “rule-out,” test is intended to identify individuals unlikely to have AD pathology, typically in low-prevalence settings where a negative result is expected to have high negative predictive value (NPV).^17^ By contrast, a confirmatory test is used in higher pretest probability settings, where a positive result has sufficiently high positive predictive value (PPV) to support aetiologic attribution without further testing. However, the value of a rule-out strategy in primary care requires careful interpretation. In low-prevalence populations, a high NPV will reflect the underlying rarity of amyloid pathology as much as test discrimination. The central question is therefore whether a negative result is being applied to the right clinical population and for the right purpose.

Here, the public health distinction between screening, case finding, and diagnostic testing becomes important. Screening tests asymptomatic populations to identify disease earlier^18^; case finding identifies people at increased risk^19^; diagnostic testing is undertaken in symptomatic individuals to establish the presence or absence of disease. In theory, an AD BBM used after adequate clinical assessment in a symptomatic person remains diagnostic. In practice, when introduced into primary care populations with broad, nonspecific, or incompletely characterized cognitive complaints, its function may drift towards case-finding or opportunistic screening.

Foley et al.^20^ account of the “current” vs “future” primary care pathway is instructive here. It shows how the therapeutic era may encourage earlier biomarker-oriented investigation and referral, even when symptoms are mild or diagnostically uncertain. This risks AD BBMs being used not as carefully sequenced diagnostic adjuncts but as tools for premature biological classification.

For this reason, the principal risk in primary care is not simply imperfect test performance, but category drift. If AD BBMs are applied in populations with poorly specified pretest diagnostic probability, and before sufficient clinical anchoring has occurred, they risk moving from targeted diagnostic use towards quasi-screening without the conceptual, evidentiary, or governance safeguards ordinarily required of screening programmes.^21^ In this setting, the test result may be numerically valid yet clinically misplaced. The category error is therefore twofold: first, the test may be applied outside an appropriately defined clinical population; second, the resulting biological signal may be given an interpretive weight not justified by the preceding clinical assessment.

For these reasons, pretest probability should not be treated as a purely statistical precondition for AD BBM use but as an extension of clinical anchoring itself. In primary care, the more weakly specified the syndrome, the less defined the pretest probability, and the greater the risk that a purported rule-out strategy functions less as diagnosis and more as opportunistic case finding.

## The Impact of a “Rule-Out” AD BBM Test in Primary Care

The value of a rule-out test lies in whether its result safely changes subsequent management. In practice, a negative result would need to support at least 1 of 3 consequences: avoidance of referral, delayed referral, or lower-intensity referral.

Our critique is framed within a specific, and in many settings prevailing, care pathway: once cognitive impairment has been objectively established and major reversible causes have been excluded in primary care, patients are referred for specialist assessment leading to formal diagnosis. This assessment addresses *both* suspected AD and alternative nonreversible causes of cognitive decline.^8,22^

In this pathway, a negative rule-out result does not change management in primary care. Once objective cognitive impairment has been established and reversible causes reasonably excluded, the patient still requires specialist etiological assessment. A negative AD BBM result may lower the likelihood that AD pathology is contributing but does not remove the need to investigate alternative irreversible causes of cognitive decline.

Accordingly, a negative result in primary care should not: prevent referral, because the patient still requires specialist diagnosis of the cause(s) of decline; delay referral, because timely assessment is relevant not only for possible AD but also for vascular, Lewy body, frontotemporal, mixed and other dementias; substantially downgrade referral intensity, because these alternative etiologies are investigated within the same downstream service. Under these assumptions, the operational leverage of a primary care rule-out test is limited.

We therefore do not propose rule-out testing as a preferred primary care strategy. Rather, we use it as a critical example of a plausible but potentially misleading implementation model. The intended use of the 181P plasma assay illustrates this tension.^4^ Although negative results are described as being consistent with corresponding amyloid PET findings, further evaluation for alternative causes of cognitive decline remains recommended, and the assay is not intended for patients already referred to specialist care. The test therefore informs only 1 biological component of a broader etiologic problem.

This limitation is amplified by the breadth of the intended-use population, including individuals with subjective complaints and lower pretest probability of amyloid pathology.^4^ In such populations, the appeal of a rule-out strategy is strengthened by high NPV, but the positive arm may become noisy and clinically difficult to interpret.

The mismatch is further accentuated by mixed pathology^23^: in specialist settings, the question is rarely “Alzheimer” vs “not Alzheimer,” but which pathologic processes are contributing to the presentation and which are most clinically relevant. A negative AD BBM cannot resolve that broader problem, just as a positive result cannot establish that AD pathology is the principal driver of symptoms.

A rule-out strategy could, in principle, add value under different pathway conditions: if primary care had sufficient capacity for longitudinal diagnostic workup and management of non-AD causes without routine specialist referral; if specialist services were partitioned such that a negative AD BBM meaningfully redirected patients into different pathways; or if clear protocols allowed test-negative, low-risk cases to remain in primary care with structured follow-up and defined re-escalation criteria. In such settings, a negative result might alter management. The problem is not that rule-out logic is intrinsically unsound, but that these conditions are not yet characteristic of many real-world systems.

For these reasons, the impact of a routine rule-out AD BBM in primary care is likely to be small. A high NPV may reduce confidence in amyloid pathology, but if the patient still requires specialist assessment, the test has not meaningfully changed the pathway. The central issue is therefore not whether the assay can exclude amyloid pathology with sufficient confidence, but whether doing so changes what the health system must do next. Where the answer is no, the value proposition of routine rule-out use is weak.

## Modelling the Downstream Impact of a Rule-Out Test in Primary Care

If a rule-out AD BBM is to be implemented in primary care, its value must be judged not solely on test performance but also by its effects on the care pathway. In low-prevalence settings, even a test that meets proposed triage performance thresholds may generate a substantial number of positive results, with important downstream consequences.^24^ To illustrate this, we model 2 hypothetical primary care scenarios (n = 1,000) with AD pathology prevalences of 20% and 15%, applying the AA 2025 Clinical Practice Guideline thresholds for a triage blood-based biomarker test (≥90% sensitivity and ≥75% specificity), as shown in Tables 1 and 2.^7^

**Table 1:** Illustrative Primary Care Scenario Assuming 20% AD Pathology Prevalence, 90% Sensitivity, and 75% Specificity in 1,000 Tested Individuals

	AD pathology present	AD pathology absent	Total
Test positive	180 true positives	200 false positives	380
Test negative	20 false negatives	600 true negatives	620
Total	200	800	1,000

Abbreviations: AD = Alzheimer disease; NPV = negative predictive value; PPV = positive predictive value.

Under these assumptions, PPV = 180/380 = 47.4%; NPV = 600/620 = 96.8%. The model is illustrative and is intended to show how test performance interacts with disease prevalence in a hypothetical primary care population.

**Table 2:** Illustrative Primary Care Scenario Assuming 15% AD Pathology Prevalence, 90% Sensitivity, and 75% Specificity in 1,000 Tested Individuals

	AD pathology present	AD pathology absent	Total
Test positive	135 true positives	213 false positives	348
Test negative	15 false negatives	637 true negatives	652
Total	150	850	1,000

Abbreviations: AD = Alzheimer disease; NPV = negative predictive value; PPV = positive predictive value.

Under these assumptions, PPV = 135/348 = 38.8%; NPV = 637/652 = 97.7%. The model is illustrative and is intended to show how test performance interacts with disease prevalence in a hypothetical primary care population.

Under these assumptions, the proportion of tested individuals generating a false positive test result is substantial. At a prevalence of 20%, 200 of 1,000 tested individuals would be false positive, yielding a PPV of 47.4%; at a prevalence of 15%, 213 of 1,000 would be false positive, with a PPV of only 38.8%. The positive arm is not a secondary inconvenience of rule-out testing; it is a predictable consequence of deploying a test with imperfect specificity in a low-prevalence population and must be included in any assessment of pathway utility. Thus, in a low-prevalence primary care population, a triage test that performs well against sensitivity and specificity thresholds can still generate a large downstream group in whom the initial positive signal does not correspond to true AD pathology.

This matters because, as argued above, the negative arm of the pathway may not reduce the need for onwards referral, whereas the positive arm is likely to trigger onward referral, confirmatory testing, or both. The resulting asymmetry is important: the test may not safely remove enough patients from the specialist pathway to offset the additional demand generated by false-positive referrals.

When projected onto current diagnostic pathways, this pattern is likely to create bottlenecks, particularly in systems with limited access to CSF, amyloid PET, or specialist services required to formalize an AD diagnosis.^25^ The intended use of the 181P assay reflects this limitation: a positive result may not be consistent with a positive amyloid PET scan and requires further investigation, while a negative result lowers the likelihood of amyloid pathology but does not obviate evaluation for other causes of cognitive decline. The assay is therefore positioned as a preliminary sorting tool rather than as a stand-alone solution to diagnostic decision-making.

## Actionability and Clinical Benefit

A further question is whether the additional biological information generated by this strategy is meaningfully actionable for the person being tested. Here, clinical utility should be understood as evidence that use of the biomarker result improves patient-relevant outcomes.^26^ Whether such utility exists depends on intended use, relevant end points, magnitude of benefit, and the service context in which the result is acted upon.^9^

An AD BBM detects AD-related pathology; it does not in itself establish a clinical diagnosis, determine the dominant driver of symptoms, or identify treatment eligibility. Although some patients with a positive test in primary care might reach specialist assessment and anti-amyloid treatment (AAT) pathways more quickly, any benefit remains conditional on specialist capacity, confirmatory testing infrastructure, and restrictive eligibility criteria.^24^ Clinical utility also remains uncertain for patients who are not candidates for AAT: a positive AD BBM does not determine access to symptomatic treatments (i.e., cholinesterase inhibitors or memantine)—initiated only after the clinical syndrome has been established and appropriately subtyped, and evidence remains limited that biological characterization alone improves psychosocial interventions, risk factor management, or routine postdiagnostic support.^27^ Moreover, any benefit depends on service ability to translate earlier biological information into meaningful care. In this respect, the actionability of a positive primary care biomarker result may be narrower than the suggested strategic importance of earlier detection.^28^

A distinct but related future use of AD BBMs is monitoring biological response to disease-modifying therapies. Longitudinal changes in plasma p-tau concentration, and potentially other markers of neurodegeneration and neuroinflammation may provide complementary information on treatment-associated biological change and could reduce reliance on more invasive or resource-intensive modalities in selected settings. However, treatment monitoring represents a different context of use from primary care diagnostic triage.

## Fast-Track Prioritization

If routine rule-out use in primary care has limited pathway value, a narrower implementation model might still be considered: selective fast-track prioritization. Here, an AD BBM would identify a subgroup for faster access to an Alzheimer-focused confirmatory and treatment pathway. This differs from rule-out testing: its purpose is not referral substitution but queue reordering.

AATs have introduced a time-sensitive clinical logic in which earlier identification of potentially eligible patients may appear advantageous. However, this does not in itself justify fast-tracking all individuals with a positive AD BBM result. The key issue is enrichment. A fast-track strategy is only defensible if a positive test meaningfully increases the probability that the patient will prove to have clinically relevant AD pathology and benefit from expedited specialist work-up. Yet the positive arm of a primary care AD BBM may be too imprecise to support such use as a stand-alone prioritization signal. In the P181 study, PPV was only 22.4%, indicating that many positive tests would not correspond to true amyloid positivity.^4^ A fast-track strategy would therefore require a biomarker with substantially higher PPV, closer to that shown by plasma p-tau217 in specialist settings, although this has yet to be validated in primary care settings.

This has important implications for queue management. Fast-tracking all positive results would not simply accelerate access for a biologically enriched subgroup; it would also expedite a substantial number of patients who may ultimately prove not to have amyloid pathology, while potentially displacing others with non-AD but equally time-sensitive causes of cognitive decline. In systems with finite specialist capacity, confirmatory biomarker bottlenecks, and long waiting lists, the question is whether positive tests can identify patients with sufficient precision to justify preferential access over others.

Moreover, eligibility for AAT depends on a broader set of criteria, including clinical phenotype, disease stage, comorbidity profile, MRI findings, safety considerations, and local regulatory or reimbursement conditions. A positive AD BBM result in primary care therefore remains several steps removed from therapeutic actionability.

For fast-track prioritization to be considered, a more defensible model would require a holistic assessment strategy combining biomarkers with clinical phenotype, stage and other features that increase the likelihood that patients are both biologically positive and clinically appropriate for expedited treatment. In this sense, BBMs might contribute to prioritization, but are unlikely to be sufficient as a stand-alone gatekeeping tool.

## Conclusion

AD BBMs offer a more accessible means of accessing information about underlying AD pathology. However, the appeal of a blood test, compared with more resource-intensive modalities such as CSF or PET, should not obscure the clinical complexity of appropriate use. Careful consideration is required to ensure that BBMs are used at the right point in the diagnostic assessment and wider care pathway.

Our analysis suggests that routine, nonselective use of AD BBMs in primary care is not yet justified. In many healthcare systems, primary care usually serves as an initial stage of assessment before onward specialist referral. Patients with established cognitive impairment will often still require specialist aetiologic evaluation regardless of a negative result, limiting the value of a rule-out strategy. Meanwhile, false-positive results may increase referrals and confirmatory testing demands in already constrained systems, without clear material benefit for patient outcomes.

Primary care should not be expected to shoulder the full interpretive burden of AD BBMs. Distinguishing subjective cognitive concerns from MCI, estimating pretest probability, recognizing alternative neurodegenerative or vascular causes, and communicating uncertain biological results, all require time, expertise, and access to downstream services that are not uniformly available in primary care. This does not preclude primary care use in principle, but any such implementation should be selective, clinically anchored, and pathway-dependent. Before broader deployment, the purpose of testing, target population, downstream actions, and the service conditions required for safe, equitable, clinically useful and cost-effective use must be clearly defined. Conditions for responsible implementation of AD BBMs in primary care are summarized in the Figure.

**Figure:**
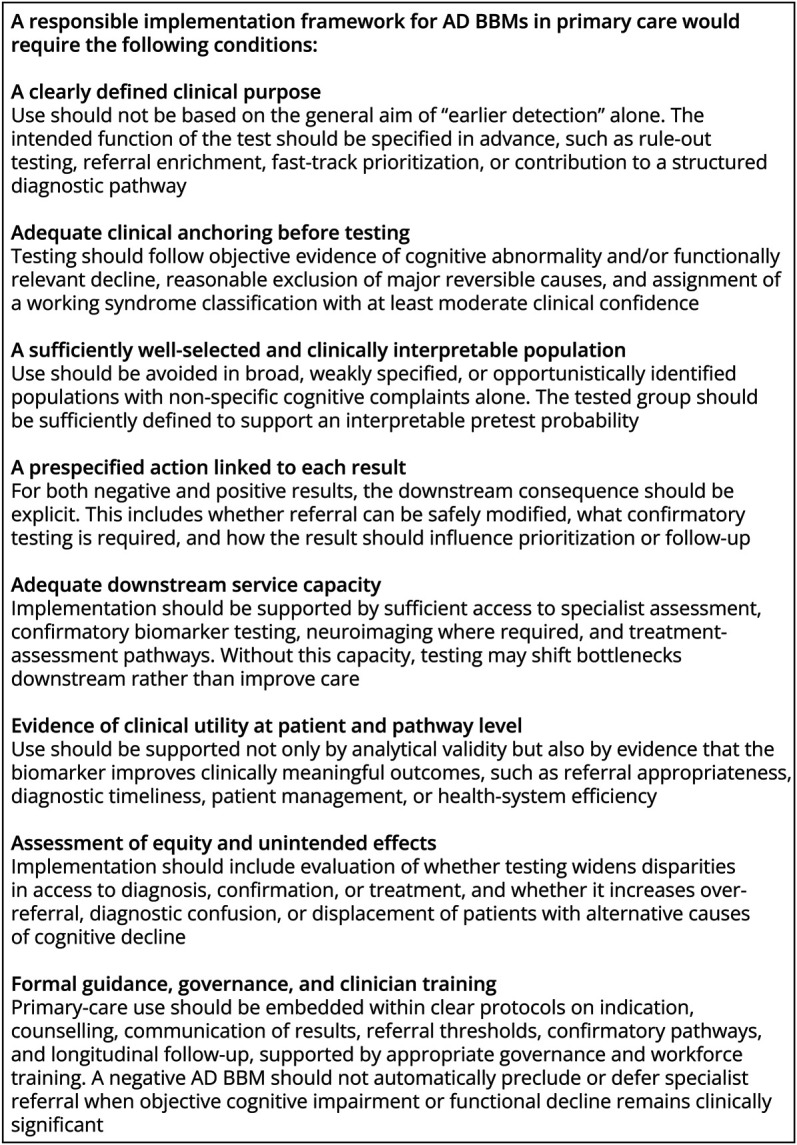
Conditions for Responsible Implementation of AD BBMs in Primary Care AD = Alzheimer disease; BBMs = blood-based biomarkers.

Further research, together with engagement of policymakers and public health bodies, is needed to define the appropriate role of AD BBMs in primary care. Any such deployment should demonstrate acceptable cost-effectiveness at the health-system level, consistent with the principles used in technology appraisal in publicly funded settings.^29^ Related evaluative frameworks are already being applied to AD BBM use in specialist care.^30^ Clear guidance on the indications and conditions for use in primary care will also be required. Until such evidence, guidance, and infrastructure are in place, caution is warranted—because responsible implementation requires not only an accurate test, but also a clinically coherent use case and a health system able to act on the result wisely.

## Glossary

AA: Alzheimer's Association
AAT: antiamyloid treatment
AD: Alzheimer disease
BBM: blood-based biomarker
FDA: US Food and Drug Administration
MCI: mild cognitive impairment
NPV: negative predictive value
PPV: positive predictive value
